# Identification and functional characterization of the miRNA-gene regulatory network in chronic myeloid leukemia lineage negative cells

**DOI:** 10.1038/srep32493

**Published:** 2016-09-02

**Authors:** S. Agatheeswaran, N. C. Pattnayak, S. Chakraborty

**Affiliations:** 1 Institute of Life Sciences, Nalco Square, Bhubaneswar, Odisha, India; 2 Department of Clinical Haematology, SCB Medical College, Cuttack, Odisha, India

## Abstract

Chronic myeloid leukemia (CML) is maintained by leukemic stem cells (LSCs) which are resistant to the existing TKI therapy. Hence a better understanding of the CML LSCs is necessary to eradicate these cells and achieve complete cure. Using the miRNA-gene interaction networks from the CML lin(−) cells we identified a set of up/down-regulated miRNAs and corresponding target genes. Association studies (Pearson correlation) from the miRNA and gene expression data showed that miR-1469 and miR-1972 have significantly higher number of target genes, 75 and 50 respectively. We observed that miR-1972 induces G2-M cell cycle arrest and miR-1469 moderately arrested G1 cell cycle when overexpressed in KCL22 cells. We have earlier shown that a combination of imatinib and JAK inhibitor I can significantly bring down the proliferation of CML lineage negative cells. Here we observed that imatinib and JAK inhibitor I combination restored the expression pattern of the down-regulated miRNAs in primary CML lin(−) cells. Thus effective manipulation of the deregulated miRNAs can restore the miRNA-mRNA networks that can efficiently inhibit CML stem and progenitor cells and alleviate the disease.

Chronic myeloid leukemia (CML) is caused by the constitutively active BCR-ABL tyrosine kinase which is a product of the Philadelphia chromosome (t (9; 22)). CML progresses from a prolonged chronic phase (CML-CP); characterized by an accumulation of apparently normal neutrophils, to a blast crisis phase (CML-BC) characterized by a clonal expansion of differentiation-arrested myeloid or lymphoid precursor cells^1^. CML is a stem cell disorder and the chronic phase of CML is propagated by a small fraction of Ph+ hematopoietic stem cells (HSC) (reviewed in ref. 2). It was reported earlier that the granulocyte-macrophage progenitor population acquires stem cell-like properties during CML blast crisis^3^. Imatinib, a small molecule BCR-ABL specific tyrosine kinase inhibitor (TKI), is the first-line of therapy for CML and helps to achieve a complete cytogenetic response (CCR) in more than 80% of the patients^4^. In spite of achieving CCR, many patients have BCR-ABL transcripts detectable by reverse-transcriptase polymerase chain reaction (RT-PCR) which suggests that imatinib fails to eradicate the leukemic stem cells in the bone marrow^5^. Consistent with this, presence of residual BCR-ABL positive CD34+ progenitors were also reported to be present in most of the CCR cases^6^. It was observed earlier that lin(−) CD34+ population, which includes HSCs and progenitors, were resistant to imatinib mediated cell death in the presence of growth factors^7^,^8^. Hence, a better understanding of the CML stem and progenitor cells is required to target and eliminate these cells.

MicroRNAs (miRNAs) are endogenous, ~22 nucleotide length small RNA molecules that negatively regulate the gene expression by directly targeting the 3′ UTR of mRNAs^9^. As miRNAs are a part of the central dogma they control a broad range of biological functions like proliferation, differentiation, apoptosis, etc.^10^. A set of miRNAs which are expressed in the hematopoietic cells play a significant role in lineage commitment and differentiation^11^. miRNA expression is deregulated in cancer cells compared to the corresponding normal tissues and they are successfully used to classify the subtypes of poorly differentiated tumours in which mRNA profiles failed to classify correctly^12^. Expression pattern of a panel of 157 miRNAs were tested in mononuclear and CD34+ cells of CML patients which showed that miR-10a was significantly downregulated in CML CD34^+^ cells that results in USF2-mediated increased cell growth^13^. Also, it was observed that the downregulation of miR-328 in CML-BC CD34+ cells favours the hnRNP E2 mediated translation inhibition of C/EBPα mRNA that results in differentiation arrested myeloid cells^14^. Given that a single miRNA can control a set of target genes and each gene can be targeted by multiple miRNAs we decided to identify the complex miRNA–gene regulatory networks present in the CML lin(−) cells which may help to delineate the disease further.

## Results

### Identification of differentially expressed miRNAs and genes

**Figure 1:**
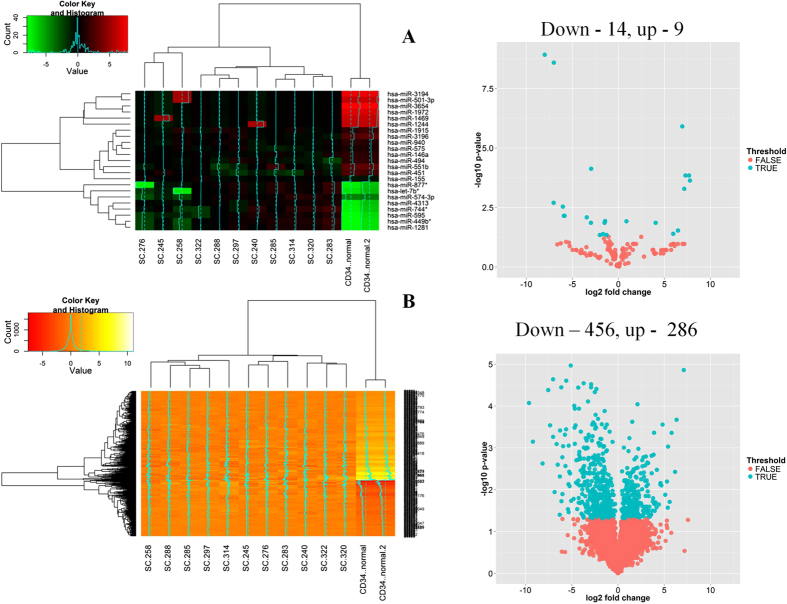
(A) Differentially expressed miRNA and (B) Genes in CML lin(−)cells are calculated by student t-test (p (corr.) ≤0.05) and represented in a heatmap (left side) and volcano plot to represent the statistical significance (right side). Log transformed normalized values of differentially expressed miRNAs and genes are represented in heat map. Volcano plot represents the statistics calculated between normal and CML group; the statistically significant entitites are highlighted in blue.

CML stem cells are known to reside in the lin(−) CD34^+^CD38^−^ population and recently it was reported that the progenitor cells acquire stem cell properties which results in the progression of the disease^3^. In this study, we have used the lin(−) population which includes the stem and progenitor cells. The lin(−) population was purified from the mononuclear cells isolated from the bone marrow of naive CML cases using the CML debulking reagent (negative selection) which contains anti-CD15 antibodies apart from the other lineage-specific markers. Approximately, 78% (mean over 11 samples) of the CML lin(−) cells were positive for CD34 surface marker compared to 34% in normal lin(−) cells (Supplementary Table 1, Supplementary Fig. 1). Our data is concurrent with the earlier reports which suggest that higher levels of CD34^+^ cells are present in CML patients. However, as lower percentage of CD34^+^ cells (34%) were isolated from normal mononuclear cells by using the debulking kit, we used purified CD34^+^ cells from Stem Cell Technologies, Canada. RNA isolated from the lineage negative cells of CML and purified CD34+ cells were processed as per the manufacturer’s instruction and hybridized to the Human miRNA 8 × 60k arrays and the Human mRNA GXP 8 × 60k V3 arrays. The differentially expressed miRNAs and genes between purified CD34^+^ and CML CD34^+^ cells were calculated by using unpaired Student’s t-test. To remove the false positive entities, we applied Benjamini and Hochberg correction with a corrected p value of ≤0.05. A total of 23 human miRNAs (Fig. 1A) and 742 genes (Fig. 1B) having Entrez ID were enriched by the analysis. Enriched (up and down) miRNAs and genes formed 2 clusters each, one consisted of up-regulated entities and the other with down-regulated entities. These results suggest that a significant number of miRNAs and genes are deregulated in CML lin(−) cells.

### Generating miRNA-gene networks from the array data

**Figure 2:**
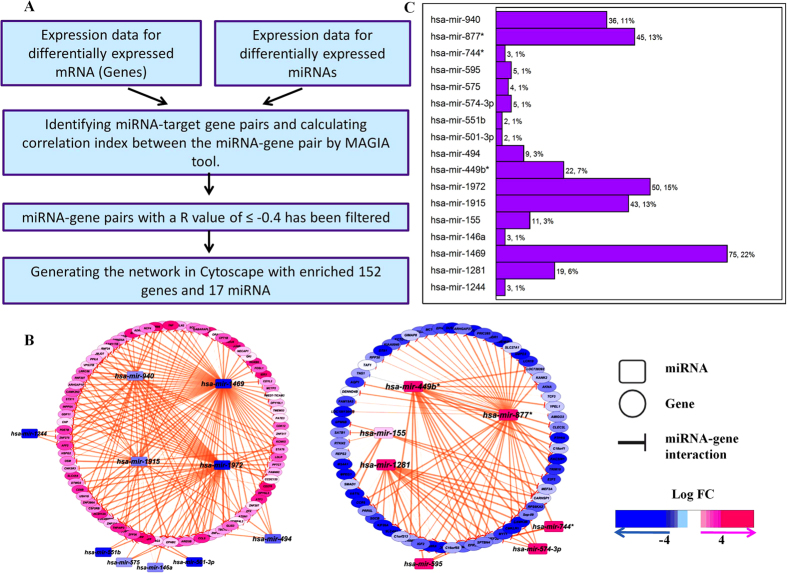
(**A)** miRNA-gene network workflow (**B**). MiRNA-gene networks generated by using MAGIA online tool and visualized in Cytoscape. **(C**) Enriched miRNA with total target genes (percentages are given in round figures).

Often miRNAs negatively regulate the gene function by directly targeting the 3′UTR of the protein-coding genes^9^. Using computational algorithms, it was observed that more than 30% of the human genes are potential targets of miRNAs^15^. To identify the miRNA-target gene networks and association between them, we used the MAGIA online tool. MAGIA uses target prediction algorithms (along with Boolean combinations) and integrates the miRNA-target gene data with the functional measures (correlation, mutual information, etc.) of the expression data. We used the expression profiles of 23 miRNAs and 742 genes which were enriched by Student’s t-test, for MAGIA analysis. We have combined the results of two different target prediction algorithms: miRanda which is based on sequence similarity and TargetScan which identifies sequence similarity with conservation (Fig. 2A). Pearson correlation (r value) index between each predicted miRNA – gene pair was calculated and the pairs which showed an r value of ≤−0.4 were selected for further analysis. The network consists of 152 genes and 17 miRNAs and showed 337 interactions in between them after clearing the above parameters. miRNA-gene interactions were visualized as a network in Cytoscape (Fig. 2B). The nodes (miRNA or gene) are connected by edges (negative interaction between miRNA to target gene). The node colour, which corresponds to the relative log fold change value of a particular gene or miRNA, was represented as pink for up-regulated ones and blue for down-regulated ones. The widths of the edge represent the q value (significance) of the association and are presented in an ascending scale. The resulting network consists of two main components; the first component has a set of down-regulated miRNAs with their up-regulated targets (Fig. 2B, left) while the second one is vice versa (Fig. 2B, right). Among the down-regulated miRNAs, miR-494, miR-940, miR-1469, miR-1915 & miR-1972 were found to have significantly higher number of target genes with an extensive overlap between the target genes. Among the up-regulated miRNAs, miR-449*, miR-877* and miR-1281 were found to have more number of target genes. Overall, we found that miR-1469, miR-1972, miR-877* and miR-1915 have most number of targets, 75 (22%), 50 (15%), 45 (13%) and 43 (~13%) respectively (Fig. 2C). Down-regulated miRNAs were found to control more number of target genes compared to the upregulated miRNAs. Gene ontology (GO) analysis of the network enriched 152 target genes showed that most of them are involved in the genome maintenance, transcription and lineage commitment (cellular fate and organization) (Supplementary Fig. 2). Out of the network enriched miRNAs, miR-1469 and miR-1972 were found to have the highest number of potential targets which might play a significant role in CML.

### Validation of the differentially expressed miRNAs in archived samples

**Figure 3:**
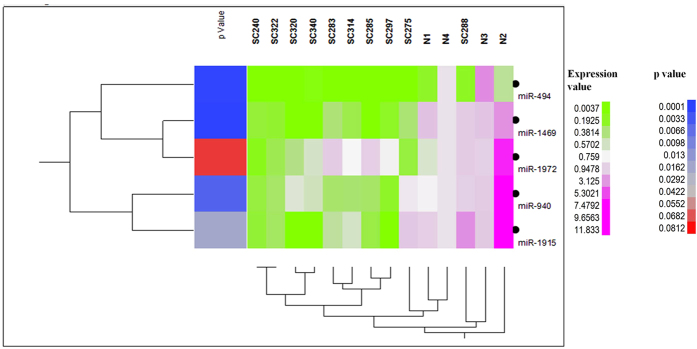
Real time PCR validation of significantly enriched miRNA from the network. Statistical significances (p values) are represented in blue to red scale. Relative expression (2^−ΔCt^) values were used for the heat map and statistical analysis.

We have selected a set of miRNAs, (miR-494, miR-940, miR-1469, miR-1915, miR-1972, miR-155, miR-877*, miR-449* and miR-1281) based on the significant associations in the network, for real-time PCR validation in the archived samples. We were not able to detect the expression of miR-877* and 449* by real-time PCR. Although the expression of miR-155 and miR-1281 were found to be up regulated, however it was non-significant when compared to the normal (Supplementary Fig. 3A,B). Statistical significance between the normal and CML samples were calculated by using Student’s t-test for the rest of the miRNAs. The data along with the p values is represented as a heatmap (Fig. 3). Statistical significances (p values) are also represented in the heatmap in a blue to red ascending order. From the data it seems that all of the miRNAs have a p value of ≤0.05 except miR-1972 which showed a p value of 0.08. The real-time PCR validation result also showed that down-regulated miRNAs have significant changes compared to the up-regulated miRNAs. Lu *et al.*^12^ has also observed a global decrease in miRNAs in human cancers compared to the tissue matched controls. Another study has also reported lower expression of miRNAs in human cancer cell lines, NCI-60 panel, compared to the corresponding normal tissues^16^. Decreased global miRNA expression pattern may reflect the state of cellular differentiation since miRNAs can prevent the cell division and drive the cellular differentiation^12^. Hence it may be assumed that down-regulated miRNAs might control cancer pathogenesis and progression.

### Differential expression of enriched miRNAs in imatinib treated primary CML stem and progenitor cells

**Figure 4:**
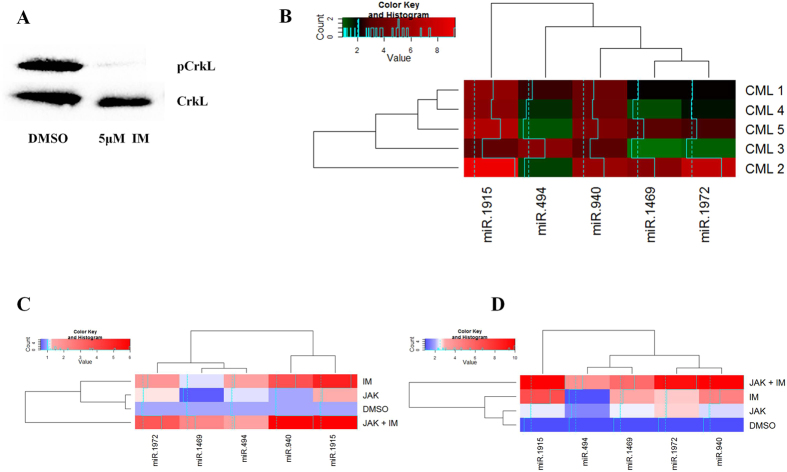
(**A**) Crkl and pCrkl expression in imatinib treated primary CML lin(−) cells. (**B**) Real time PCR quantification (fold change) of validated miRNAs in imatinib treated primary CML lin(−) cells with respect to the DMSO control. Expression pattern (fold change) of network enriched miRNAs in CML lin(−) cells treated with imatinib, JAK inhibitor I and combination of imatinib and JAK inhibitor I along with DMSO control. (**C**) CML patient 1 (**D**) CML patient 2.

Since BCR-ABL is the hallmark oncogene in chronic phase and specific tyrosine kinase inhibitors are available, we hypothesized that blocking BCR-ABL kinase activity with imatinib might resume the miRNA levels. To validate the hypothesis further, we treated five independent primary CML lin(−) cells with imatinib. Earlier reports suggest that primary lin(−) cells are resistant to imatinib even at higher concentrations^7^,^8^. So we have used 5 μM imatinib for 48 hrs which reduced the cell proliferation but failed to induce cell death in primary cells in the presence of growth factors (Supplementary Fig. 4A,B). No change in cell cycle pattern was observed (data not shown). Imatinib efficiently inhibited BCR-ABL kinase activity as phospho-CrkL levels were significantly reduced compared to the total CrkL in the imatinib treated cells (Fig. 4A). This phenomenon was already observed by others and it was proposed that these cells do not depend on BCR-ABL kinase activity for their survival and growth factors can stimulate alternative survival signaling pathways^7^,^8^,^17^,^18^. Among the 5 down-regulated miRNAs, miR-940 and miR-1915 were found to be up-regulated in all the samples after imatinib treatment (Fig. 4B). But the other three miRNAs (miR-494, miR-1469 and miR-1972) were up-regulated in only 2 samples and the degree of up-regulation was also lower when compared to miR-940 and miR-1915. Thus, it appears that the BCR-ABL kinase activity controls the expression of these miRNAs and it can be partially reversed by TKI’s. Stromal cells produce some growth factors which are mainly responsible for the activation of JAK-STAT pathway apart from BCR-ABL and protect CML stem cells when exposed to TKIs^8^,^18^. We have reported earlier that a combination of 50 nm JAK inhibitor I along with 5 μM imatinib was able to inhibit CML lin(−) cells^19^. We have also shown that JAK inhibitor I treatment significantly reduced pSTAT3 protein which was not altered with imatinib treatment^19^. Here we observed that two samples when treated with a combination of imatinib and JAK inhibitor I significantly showed up regulated miR-1972 and miR-1469 expression which was not significant when imatinib was used alone. The expression of other three miRNAs (miR-494, miR-940 and miR-1915) was also significantly up regulated in imatinib and JAK inhibitor I combination when compared to imatinib (Fig. 4C,D). One of the two samples that were checked for the expression of downregulated miRs after imatinib and JAK inhibitor I treatment, was used earlier^19^ for checking the pSTAT3, STAT3, pCRKL, CRKL, cell number and BCR-ABL/BCR after imatinib and JAK inhibitor I treatment. Thus it seems that JAK inhibitor I and imatinib treatment can not only reduce cell prolifeartion but also restore the network enriched miRNA expression in CML stem and progenitor cells.

### miR1972 induces G2-M cycle arrest and miR1469 induces G1 cell cycle arrest

**Figure 5:**
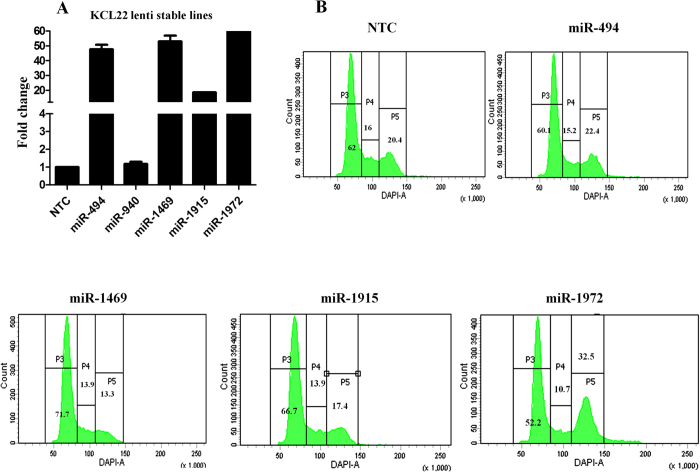
(**A**) Real time PCR data shows the over expression of miR 494, 1469, 1915 and 1972 in the respective stable lines compared to non targeting control (NTC). Fold change values were derived from technical replicates. (**B**) Cell cycle analysis in KCL22 stable lines.

To validate the functional significance of the network enriched miRNAs we developed stable lines of KCL22 overexpressing the enriched miRNAs. We selected the network enriched miRNAs, miR-494, miR-940, miR-1469, miR-1915 and miR-1972 and transduced them individually in KCL22 cells using lentiviral vectors carrying the miRNA of interest along with turbo GFP and puromycin resistant gene. We found more than 95% of the cells express turbo GFP in all the stable lines (data not shown) and the real-time PCR data showed that all the miRNAs were significantly up-regulated except miR-940 (Fig. 5A). We performed the remaining experiments without using KCL22 miR-940 stable line. As miRNA expression can prevent cell division and promote differentiation^12^, we analyzed the cell cycle pattern in the stable lines by using DAPI. The cell cycle pattern showed that miR-1972 induces a significant G2-M arrest whereas miR-1469 induces a moderate G1 arrest (Fig. 5B). We also found that the cell cycle inhibition by miR-1972 was associated with the up-regulation of p21 at both mRNA and protein levels compared to the NTC (Supplementary Fig. 5).

### MiR-1972 targets cell cycle regulator CDC25B

**Figure 6:**
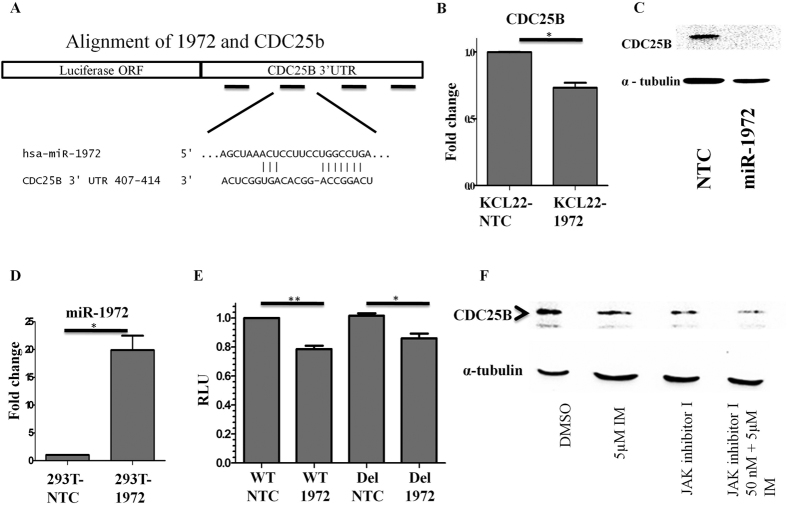
(**A**) CDC25B 3′UTR targeted by miR-1972. (**B**) mRNA and (**C**) protein expression in miR-1972 transduced KCL22 cells. (**D**) miR-1972 expression in 293T stable lines. (**E**) Relative luciferase activity of WT and mutated CDC25B 3′UTR with miR-1972. **(F**) CDC25B expression in cells treated with imatinib, JAK inhibitor I and combination of imatinib and JAK inhibitor I along with DMSO control.

Since miR-1972 induces G2-M arrest and from Fig. 2B it seems that no cell cycle regulatory genes are targeted by miR-1972, we used Target Scan database to identify the cell cycle regulators targeted by miR-1972. Among the miR-1972 targets, GO analysis identified a set of cell cycle regulators and we hypothesized that through these targets the miRNA can inhibit the cell cycle progression. Based on the TargetScan total context score we found CDC25B as a potential target since it has the lowest total context score (−0.72). The context score is the sum of the contribution of four features: site-type contribution, 3′ pairing contribution, local AU contribution and position contribution (http://www.targetscan.org/vert_50/docs/context_scores.html). Here poorly conserved sites are given the same weight as conserved sites. CDC25B is an important regulator of cell cycle progression and is overexpressed in many cancer types. Four predicted sites were observed for miR-1972 in CDC25B 3′UTR, out of which one is an 8-mer site and remaining are 7-mer-1A sites (Fig. 6A). CDC25B mRNA (Fig. 6B) and protein (Fig. 6C) expression were significantly down-regulated in miR-1972 transduced KCL22 cells. To confirm CDC25B as a direct target, we transfected the CDC25B 3′UTR construct along with the renilla control in 293T and the stable cell lines were transduced with NTC and miR-1972 (Fig. 6D). Relative luciferase activity was significantly down-regulated in miR-1972 expressing cells compared to the NTC expressing cells (Fig. 6E, bar 2 with respect to bar 1). Deletion of the 8-mer site partially relieved the repression suggesting the involvement of other sites apart from the 8-mer site (Fig. 6E, bar 4 with respect to bar 3). Thus miR-1972 can also perform as a tumour suppressor since it inhibits CDC25B expression. CDC25B expression was moderately down-regulated when CML primary cells were treated with either imatinib or JAK inhibitor I, however showed a drastic change when a combination of the drugs were used (Fig. 6F). Altogether, this data suggests that miR-1972 can block the cell cycle by directly targeting CDC25B and a combination treatment of the primary cells with imatinib and JAK inhibitor I not only up regulates the expression of miR-1972 as shown in Fig. 4C,D, but also degrades CDC25B.

## Discussion

Regardless of the success with TKIs, residual BCR-ABL positive CML cells are found in the majority of patients. A fraction of the BCR-ABL positive CD34^+^ cells residing in the bone marrow are resistant to the TKIs^6^ and the mechanisms responsible for their survival are being studied extensively^17^,^18^. The intrinsic factors like epigenetic regulators and miRNAs help the stem and progenitor cells to maintain their state and are mainly responsible for the cellular hierarchy^20^. As miRNAs are involved in hematopoiesis and can modulate differentiation^11^, it could play a significant role in leukemic stem and progenitor cells. Cell cycle regulator CDC25B, a direct target of miR-1972 was found to be significantly down-regulated in miR-1972 transduced cells. Earlier it was shown that over expression of miR-223 was able to induce granulocytic differentiation in acute promyelocytic leukemia (APL) cells^21^ and later it was observed that miR-223 targets E2F1 and inhibits cell cycle progression which results in myeloid differentiation^22^. Over expression of miR-328 which is down-regulated in the CML-BC CD34+ cells, was able to induce C/EBPα protein expression and subsequently granulopoiesis^14^.

Agirre *et al.*^13^ deduced a miRNA expression profile by testing 157 miRNAs in mononuclear cells and CD34+ cells from patients with CML and healthy controls which showed down-regulation of miR-10a, miR-150, miR-151 and up-regulation of miR-96 in CML cells. We generated the miRNA-mRNA networks using differentially expressed miRNA and mRNA from the CML lin(−) cells which can be used to study the functional role of the miRNAs in CML stem and progenitor cells. Network-based approaches provide visualization of complex biological interactions and also help to integrate the biological data derived from the different sources. The following down-regulated miRNAs, miR-494, miR-940, miR-1469, miR-1915 & miR-1972 were enriched from the miRNA-gene networks. Association studies (Pearson correlation) from the expression data showed that miR-1469 and miR-1972 has significant number of potential targets. Over expression of the miR-1469 and miR-1972 has resulted in the cell cycle arrest at G1 and G2-M stages respectively. Earlier it was proposed that global miRNA expression may reflect the state of cellular differentiation and miRNAs can prevent the cell division and drive the cellular differentiation^12^.

Recently it was reported that miRNA-1469 directly targets STAT5a and promotes lung cancer cells apoptosis^23^. We observed that the expression of STAT5A was upregulated in our array data, however it was below the cut-off value of 2-fold. Almost 100% of the mice reconstituted with BCR-ABL (p210)-transduced STAT5a (+/+) BM cells developed classic CML however only a quarter developed the disease with BCR-ABL (p210) transduced STAT5a (−/−) BM cells^24^. STAT5a attenuation also inhibits growth of CML CD34+ cells isolated from patients with acquired resistance to imatinib^25^. Thus it will be worthy to find if at all over expression of miR-1469 in CML lin- cells can down modulate the expression of STAT5a. In accordance with our data it was shown that miR-940 was downregulated in several cancers including pancreatic ductal adenocarcinoma (PDAC)^26^, hepatocellular carcinoma^27^, prostate cancer^28^, nasopharyngeal carcinoma^29^ and miR-494 were downregulated in oral cancer^30^, cervical cancer^31^, and pancreatic cancer^32^. Ectopic expression of miR-494 suppressed the cell proliferation in gastric cancer^33^ and prostate cancer cells^34^. Thus we systematically identified a group of down regulated miRNAs in CML which was also reported to be downregulated in solid tumors. Work is underway in the lab to decipher the role of each of the miRNAs in CML stem and progenitor cells.

Clinical JAK2 inhibitor ruxolitinib along with nilotinib has efficiently eliminated the BCR-ABL positive stem and progenitor cells^35^. We have also reported that imatinib and JAK inhibitor 1 combination eliminated the BCR-ABL positive lin(−) cells better than any single agent therapy^19^. Inhibition of BCR-ABL kinase with imatinib in the primary lineage negative cells failed to increase the expression level of miR-1469 and 1972. Dual inhibition of BCR-ABL and JAK kinases resulted in the up-regulation of miR-1469 and 1972 in the primary CML stem and progenitor cells accompanied with a significant reduction of CDC25B protein. It suggests that these miRNAs may function as tumor suppressors. Thus resuming miR-1972 expression levels in CML stem and progenitors by a combination treatment of imatinib and JAK inhibitor I can inhibit the cell division and reduce the proliferation. Thus agents which can activate these miRNAs, might increase the efficiency of TKIs on CML stem and progenitor cells.

## Materials and Methods

### Patient samples and cell lines

The study was approved by the institutional human ethical committee and the bone marrow samples were collected from the newly diagnosed CML patients with the written informed consent. All experiments were performed in accordance with relevant guidelines and regulations, and the Review Board of Institute of Life Sciences had approved all experiments. CML stem and progenitor cells were isolated by using CML debulking kit (14154, Stem Cell Technologies, Canada). Normal BM-derived CD34^+^ (ABM017F) cells were purchased from Stem Cell Technologies, Canada. PE tagged CD34^+^ antibody (10513) and corresponding isotype control (10311), both from Stem Cell Technologies, Canada, were used for flow cytometry. CML-BC derived KCL22 cell line was used for further *in vitro* studies.

### RNA isolation and cDNA preparation

Total RNA from the samples was isolated by using either Ambion’s miRVana kit (AM1560, Life Technologies) or Trizol (15596-018, Life Technologies, USA). DNA contamination in the total RNA was removed by DNase I (AMPD1, Sigma-Aldrich, USA) treatment. Complementary DNAs (cDNA) from the RNA samples were prepared by using Superscript cDNA synthesis kit (18080, Life Technologies, USA). High throughput miRNA cDNAs were prepared by using NCode™ miRNA First-Strand cDNA (MIRC-50, Life Technologies, USA) synthesis kit.

### Microarray

Samples with optimal RNA purity, RIN number and quantity were considered for microarray analysis. Based on the above criteria one normal CD34^+^ (in duplicate) and eleven naïve CML samples were considered for microarray experiment. For miRNA expression, Agilent’s Human miRNA 8 × 60k array was used. For mRNA expression, Agilent’s Human GXP 8 × 60k V3 array was used. Same set of samples was used for both miRNA and mRNA array hybridization. Data were extracted using Agilent’s feature extraction software. Data analysis was carried out with Genespring GX 12.0 software (Agilent Technologies Inc, Santa Clara, CA, USA). Percentile shift normalization was applied. Volcano plots, heatmaps were generated in R^36^ using ggplot2^37^ and gplots^38^.

### Networking

MiRNA - gene networks were generated by using MAGIA online tool^39^ (http://gencomp.bio.unipd.it/magia/analysis/). MiRNA-gene networks were enhanced and visualized in Cytoscape^40^.

### Real-time PCR

Real-time PCR assay was performed in Light cycler 480 II instrument with LC 480 SYBR Green master mix (4707516001) from Roche, Mannheim, Germany. Fold change values were calculated by using 2^−∆∆Ct^ method (Table 1 for primer sequences).

### Cell culture and immunoblotting

DMEM and RPMI media with 10% heat-inactivated serum were used for culturing adherent and suspension cells respectively. CML stem and progenitor cells were grown in SFEM (Serum free expansion medium, Stem Cell Technologies) supplemented with SCF (50 ng/ml), Flt3 (20 ng/ml), IL3 (20 ng/ml) and IL6 (20 ng/ml) (all from Life Technologies) as was done earlier^19^. The cytokines were added to mimic the niche environment and also to support the growth of CML CD34+ cells. Imatinib was purchased from Cayman chemicals. JAK inhibitor I was purchased from Calbiochem. pSTAT3 (9145), STAT3 (9139), CrkL (3182) and phosphor-CrkL (3181) all from Cell Signalling Technology and α-tubulin (T5168) from Sigma were used for immunoblotting.

### Lentiviral transduction and cell cycle

Lentiviral particles (SMART choice™ shMIMIC miRNAs) encoding the five network enriched miRNAs (miR-494, miR-940, miR-1469, miR-1915 & miR-1972) along with the non-targeting control (NTC) were purchased from Thermo Scientific™ and transduced into KCL22 cells according to the manufacturer’s protocol. Transduced clones were selected using puromycin (2 μg/ml). For cell cycle analysis, the miR-stable lines were labelled with 4′, 6-diamidino-2-phenylindole (DAPI) nuclear stain (Sigma-Aldrich, USA) and acquired in LSR Fortessa flow cytometer.

### Luciferase assay

CDC25B 3′UTR and the corresponding miR-1972 binding site (8-mer) deletion construct were cloned in pMIR-REPORT™. NTC and miR-1972 expressing 293T stable lines were developed by using the lentiviral system (SMART choice™). Renilla was used as a transfection control. Firefly and Renilla luciferase activity was measured 24 hrs after transfection. Luciferase assay was performed using Promega’s (Madison, WI, USA) Dual-Glo luciferase kit. The assay was repeated three times.

### Statistics

Two groups were compared using two-tailed unpaired t-test. Multiple groups were compared using one-way ANOVA. Results with a p value of ≤0.05 were considered as significant. P values represented in figures, ≤0.05=*; ≤0.01=**; ≤0.001=***.

## Additional Information

**How to cite this article**: Agatheeswaran, S. *et al.* Identification and functional characterization of the miRNA-gene regulatory network in chronic myeloid leukemia lineage negative cells. *Sci. Rep.* **6**, 32493; doi: 10.1038/srep32493 (2016).

## Supplementary Material

Supplementary Information

## Figures and Tables

**Table 1:** MiRNA specific primers.

hsa miR-494	TGAAACATACACGGGAAACCTCAA
hsa miR-1915	GGGCGACGCGGCGGGAA
hsa miR-1972	CCAGGCACAGTGGCTCAAAA
hsa miR-940	AGGGCCCCCGCTCCCCAAAA
hsa miR-1469	CGGGGCGCGGGCTCCAAA
