# Supplementary material for: Identification and functional characterization of the miRNA-gene regulatory network in chronic myeloid leukemia lineage negative cells
Source: Sci Rep. 2016 Sep 2;6:32493. doi: 10.1038/srep32493 (PMC5009428; doi:10.1038/srep32493)

Identification and functional characterization of the miRNA-gene regulatory network in chronic myeloid leukemia lineage negative cells

S. Agatheeswaran[1], N. C. Pattnayak[2#], S. Chakraborty[1*]

[1]Institute of Life Sciences, Nalco Square, Bhubaneswar, Odisha, India

[2] Lab Care and Diagnostics, Cuttack, Odisha, India

[#] Formerly at Department of Clinical Haematology, SCB Medical College, Cuttack

[*]**Corresponding author**

Soumen Chakraborty, Ph.D

Institute of Life Sciences, Nalco Square, Bhubaneswar, Odisha, India

Phone number: 91-674-2302420

Fax number: 91-674-2300728

E-mail: soumen_ils@yahoo.co.in, soumen@ils.res.in

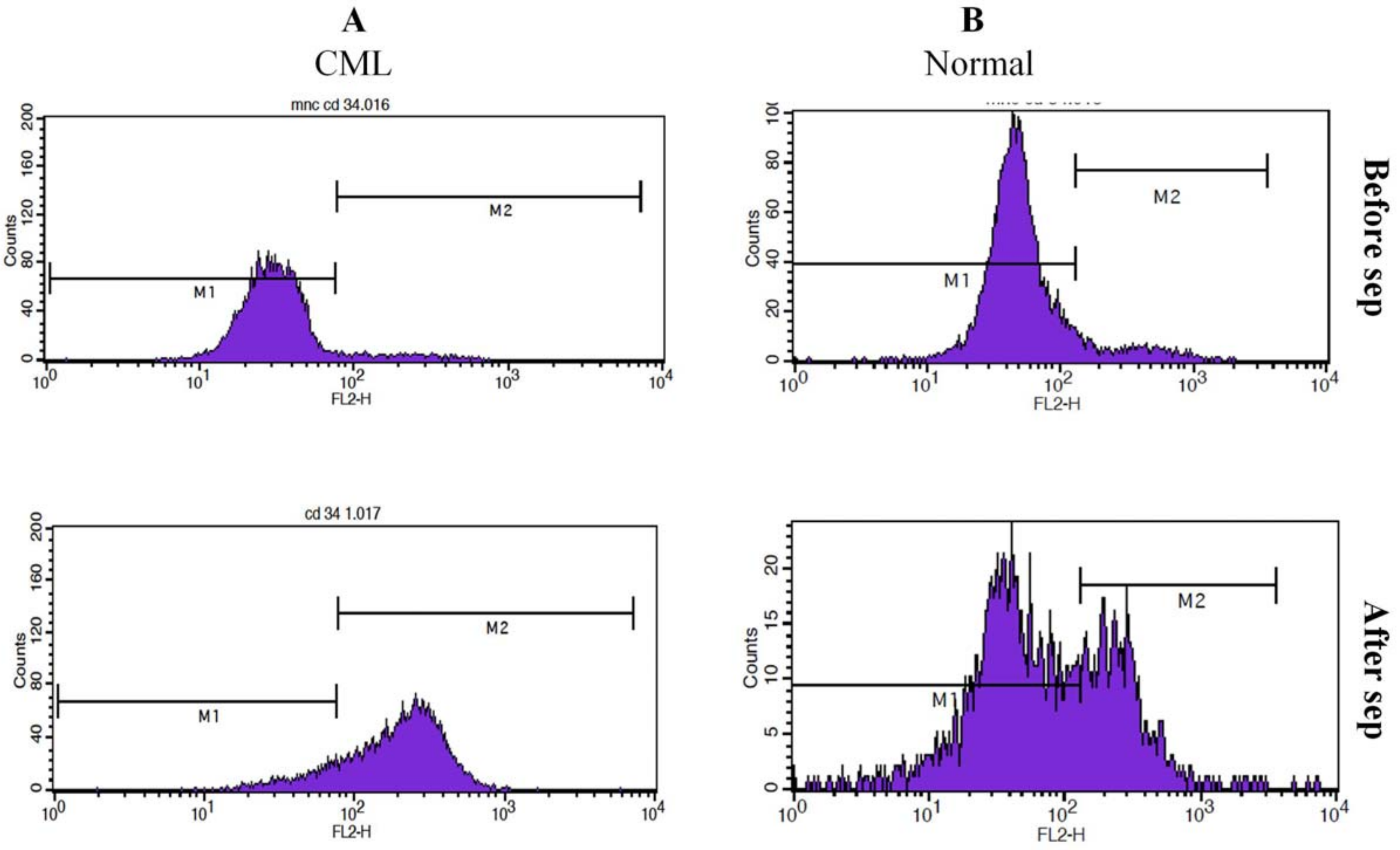

Supplementary fig. 1 CD34+ cells before and after sep in CML (a) and normal (b) lineage negative cells

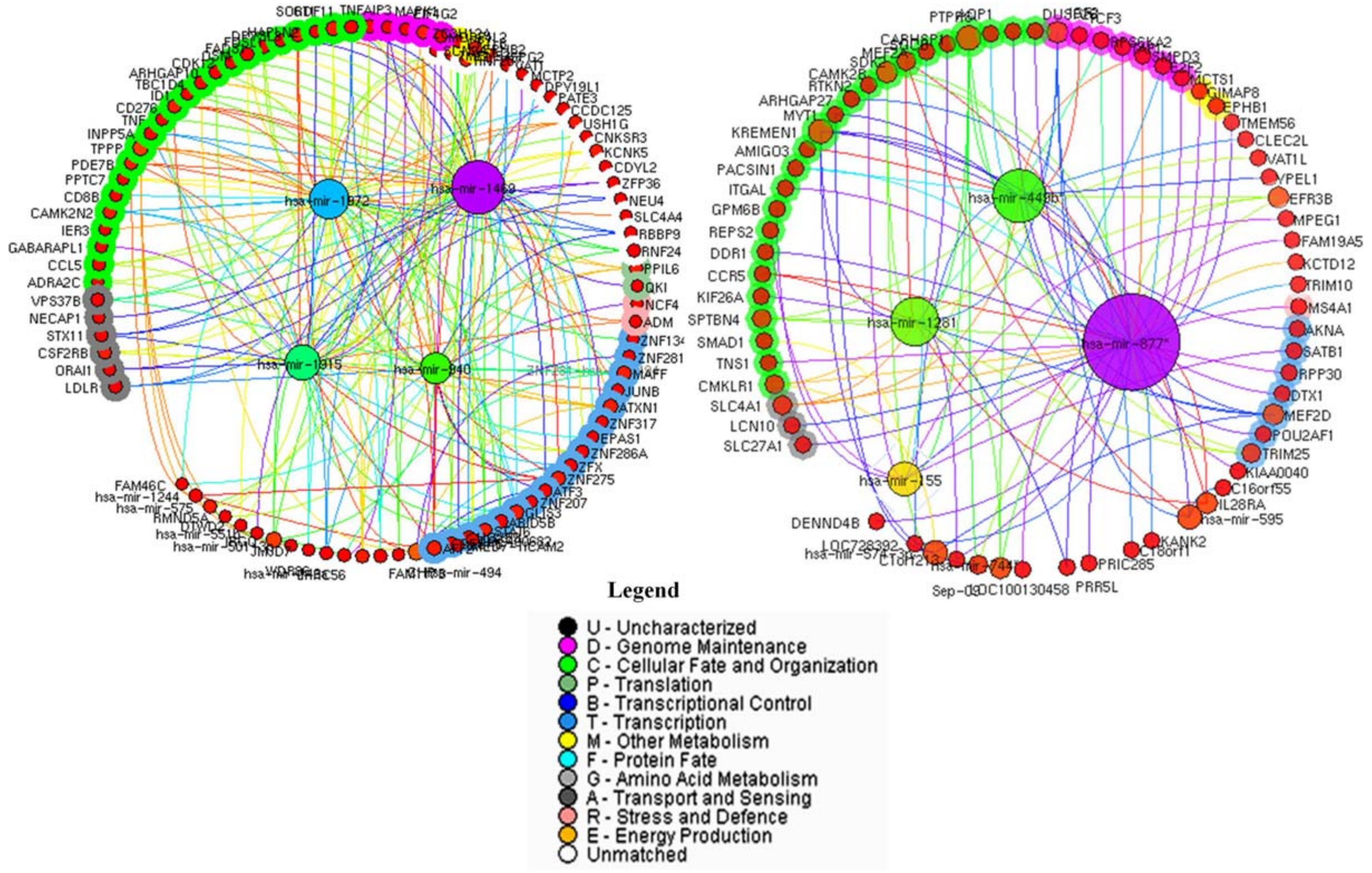

Supplementary fig. 2 miRNA-gene networks with gene ontology data. Node colour corresponds to the interactions and the node highlighting corresponds to the GO terms.

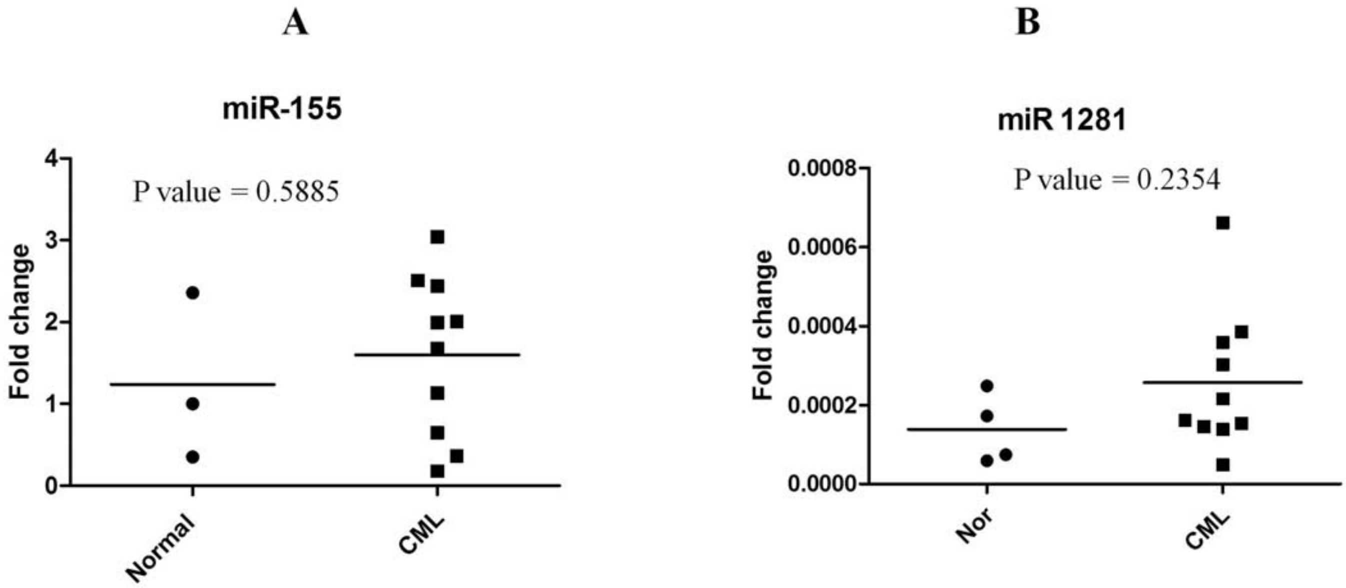

Supplementary fig. 3 Validation of network enriched up regulated miRNAs **A.** miR-155, **B.** miR-1281 in archived samples. Cross bar represents the mean of each groups.

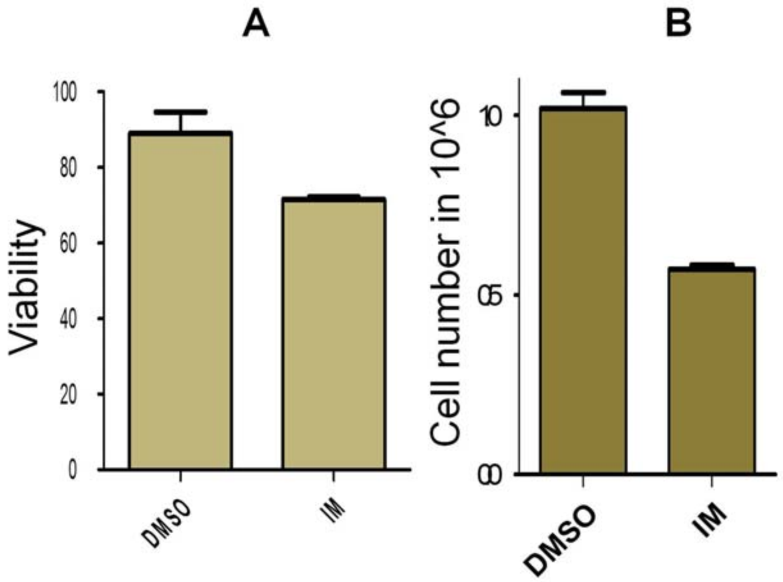

Supplementary fig. 4 Cell viability (**A**) and cell number (**B**) in imatinib treated primary lineage negative cells. Error bars represent standard deviation.

A

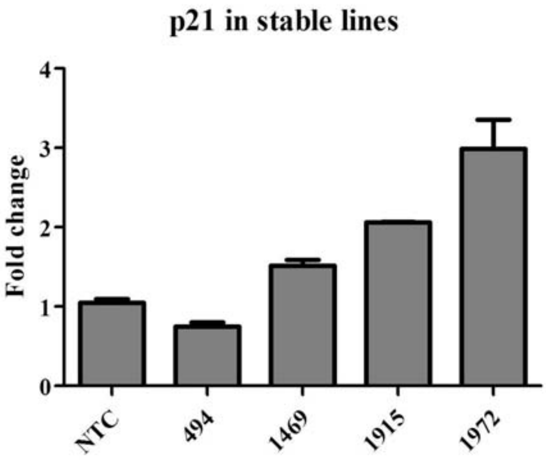

B

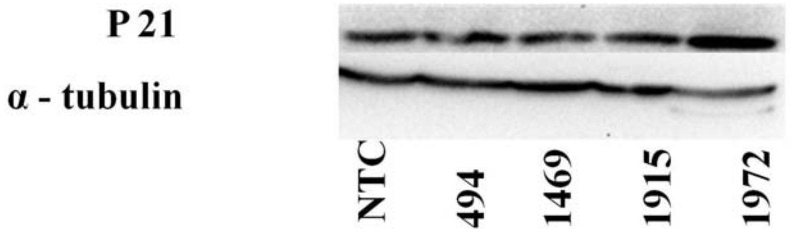

Supplementary fig. 5 Expression of p21 by real time PCR (**A**) and western blotting (**B**).

Sample	CD34+ positivity	BCR-ABL STATUS
SCCH 240 lin-	78.92	+
SCCH 245 lin-	79.83	+
SCCH 258 lin-	85.36	+
SCCH 275 lin-	72	+
SCCH 283 lin-	64	+
SCCH 285 lin-	75	+
SCCH 288 lin-	76	+
SCCH 297 lin-	84	+
SCCH 314 lin-	89	+
SCCH 320 lin-	87	+
SCCH 322 lin-	65	+
Normal $CD34^{+}$	90+	-
Normal lin-	34	-

Supplementary table 1 CD34 positivity and BCR-ABL status of normal and CML bone marrow derived lin (-) cells along with the CD 34+ normal.

Original chemidoc gel figures

4A

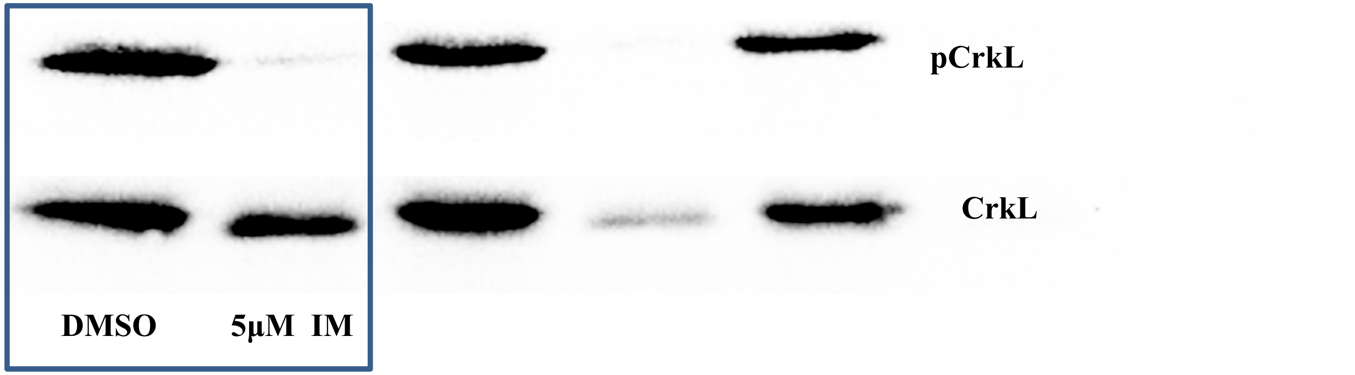

Supplement: Supplementary Information [file srep32493-s1.pdf]
